# Shifting the way we conceptualise, research and intervene childhood obesity in China and Southeast Asian countries

**DOI:** 10.1016/j.lanwpc.2025.101502

**Published:** 2025-02-26

**Authors:** Bai Li, Boyd Swinburn

**Affiliations:** a Centre for Exercise, Nutrition and Health Sciences, School for Policy Studies, University of Bristol, Bristol, UK; b School of Public Health, Guangxi Medical University, Nanning, Guangxi, China; c School of Population Health, University of Auckland, Auckland, New Zealand

China and the Southeast Asian (SEA) region respectively represent the most populous country and region in the world, and are experiencing the fastest rise in childhood overweight and obesity.^1^^,^^2^ To combat this leading public health crisis, numerous studies have been conducted in these countries to understand epidemiological patterns, risk factors and interventions of childhood obesity.^3^^,^^4^

Meanwhile, public health methods have evolved internationally as we advance to recognise that risk factors (e.g., lack of physical activity), common measures (e.g., body fatness), consequences (e.g., type 2 diabetes), and interventions (e.g., increasing physical education provision in school) of childhood obesity do not connect in a linear manner.^5^, ^6^, ^7^ Drawing on international evolvement in this field, we urge 1) a shift in the way we conceptualise, research and intervene childhood obesity in China and the SEA countries and 2) more research and policies to tackle childhood obesity simultaneously with other leading public health challenges that share common drivers and solutions.

## Shifting the way we conceptualise, research and intervene childhood obesity

Interactions can exist not only between the risk factors and outcomes, but also among the risk factors and among the different outcomes. Such complex relationships are characterised by feedback loops in which a change reinforces or balances further change, and where outcomes/outputs can act as risk factors/inputs.^8^ Research grounded in linear models of cause and effect, even at multiple levels, are unable to capture the complex, dynamic, and adaptive nature of childhood obesity.^8^ Interventions failing to recognise and address the feedback loops driving the obesogenic system can lead to unexpected consequences that weaken the desired effect of the interventions. Internationally, a growing number of studies have applied a systems approach to address the complexity of childhood obesity,^7^^,^^9^ and promising effects have been reported.^10^ However, these studies were conducted in high-income, western countries.^9^

Childhood obesity is an emergent property of complex, adaptive, global, and local systems. Therefore, tools from systems science, such as group model building to create systems maps (causal lop diagrams) and system dynamics modeling, are needed to understand the complex drivers of childhood obesity in non-western contexts. This is the first step to identify system-level leverage interventions. A recent study (SYSTAM)^11^ developed, piloted and used culturally appropriate methods from systems science to co-develop systemic nutrition interventions with policy makers from 17 governmental departments in a Chinese city of 1.05 million residents. This co-creation approach led to a 17-department Action Committee for systemic nutrition interventions being established by the municipal government to sustain multi-sector collaboration in intervention implementation, evaluation (monitoring) and optimisation. It provided China, SEA countries and other LMICs with a practical example for how to sustainably engage with decision makers in multiple sectors and departments to develop, implement and evaluate systemic childhood obesity interventions at a systemic level whereby changes in feedback loops, structures, policies, and paradigms are prioritised over programmatic approaches. It is important to note that multi-setting/sector interventions are not necessarily systemic interventions; and monitoring of systemic interventions should capture what and how system-level changes happen over time.^12^ Practical guidance is available to guide the development, implementation, evaluation, reporting, and reviewing of systemic nutrition interventions.^12^

## Moving towards systemic, synergistic interventions

The Lancet Commission report on Obesity^13^ described obesity, undernutrition and climate change as a Global Syndemic because they co-exist in time and place, negatively interact with each other and share common drivers. LMICs face the double burden of malnutrition,^14^ while fighting against the health impacts of climate change.^15^ We should encourage more research and policies that aim to address childhood undernutrition and obesity, and climate change simultaneously for synergistic effects, called double- and triple-duty actions.^13^ For example, exploring whether making infrastructure changes in cities can increase safe and active commuting between home and school while reducing air pollution, and whether enhanced regulation and support for local supply chain of school meals can help build more sustainable local food systems while reducing both undernutrition and obesity in children in LMICs.

**Fig. 1:**
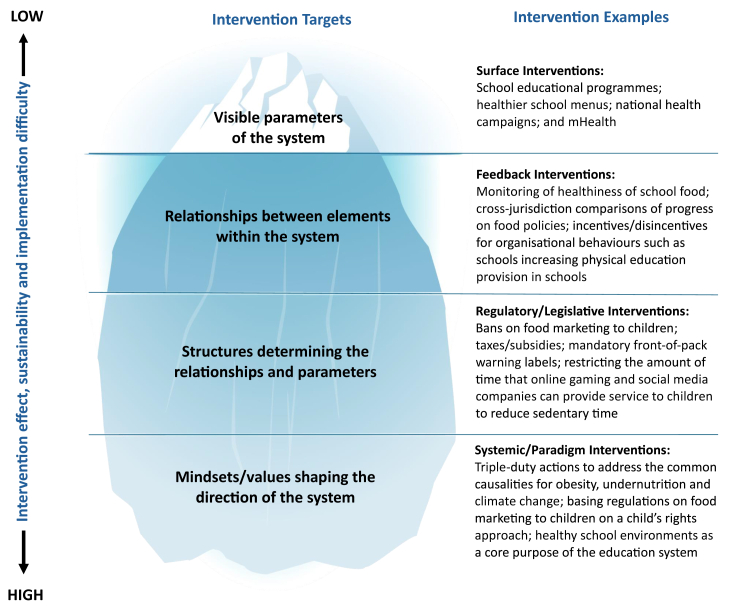
**The iceberg of childhood obesity system - conceptualising levels of interventions through systems thinking showing the interconnected layers**.

In contrast to these multi-duty actions, the increasing number of government initiatives in China and SEA countries to prevent and control childhood obesity^2^^,^^4^ have mostly been single-duty and educational in nature aiming to encourage behavioural changes by the individuals. Moreover, current interventions often target obesity, undernutrition, and different settings and age groups separately. Built on Swinburn et al.^16^ and Abson^17^ et al.'s frameworks of interventions, Fig. 1 conceptualises four levels of interventions through systems thinking. It shows that most of the systems is below the surface out of sight and the deeper the determinants, the harder they are to see and intervene on, and that the effect and sustainability of systemic and synergistic interventions (e.g., changes in mindsets to target common drivers of Global Syndemic through multi-effect actions) is greater than that of surface interventions (e.g., educational programmes and campaigns) at population level.

To effectively and efficiently combat childhood obesity in China, the SEA region and other LMICs, we urgently need transformative research and policy formulation addressing the complexity of childhood obesity, ideally with synergistic, positive effects on other public health challenges, especially undernutrition and climate change.

## Contributors

BL conceived the idea, wrote the manuscript and created the iceberg of childhood obesity system (figure). BS reviewed and edited the manuscript and the figure. Both authors proved the final version of the manuscript for submission.

## Declaration of interests

We declare no conflict of interests.
